# Expression quantitative trait loci influence DNA damage-induced apoptosis in cancer

**DOI:** 10.1186/s12864-024-11068-6

**Published:** 2024-12-02

**Authors:** Jessica Bigge, Laura L. Koebbe, Ann-Sophie Giel, Dorothea Bornholdt, Benedikt Buerfent, Pouria Dasmeh, Alexander M. Zink, Carlo Maj, Johannes Schumacher

**Affiliations:** 1 Philipps University of Marburg, Center for Human Genetics, Marburg, Germany; 2 Inventum Genetics GmbH, Mainz, Germany

**Keywords:** DNA damage, Apoptosis, Cancer, GWAS, eQTL

## Abstract

**Background:**

Genomic instability and evading apoptosis are two fundamental hallmarks of cancer and closely linked to DNA damage response (DDR). By analyzing expression quantitative trait loci (eQTL) upon cell stimulation (called exposure eQTL (e^2^QTL)) it is possible to identify context specific gene regulatory variants and connect them to oncological diseases based on genome-wide association studies (GWAS).

**Results:**

We isolate CD8^+^ T cells from 461 healthy donors and stimulate them with high doses of 5 different carcinogens to identify regulatory mechanisms of DNA damage-induced apoptosis. Across all stimuli, we find 5,373 genes to be differentially expressed, with 85% to 99% of these genes being suppressed. While upregulated genes are specific to distinct stimuli, downregulated genes are shared across conditions but exhibit enrichment in biological processes depending on the DNA damage type. Analysis of eQTL reveals 654 regulated genes across conditions. Among them, 47 genes are significant e^2^QTL, representing a fraction of 4% to 5% per stimulus. To unveil disease relevant genetic variants, we compare eQTL and e^2^QTL with GWAS risk variants. We identify gene regulatory variants for *KLF2*, *PIP4K2A*, *GPR160*, *RPS18*, *ARL17B* and *XBP1* that represent risk variants for oncological diseases.

**Conclusion:**

Our study highlights the relevance of gene regulatory variants influencing DNA damage-induced apoptosis in cancer. The results provide new insights in cellular mechanisms and corresponding genes contributing to inter-individual effects in cancer development.

**Supplementary Information:**

The online version contains supplementary material available at 10.1186/s12864-024-11068-6.

## Background

The DNA damage response (DDR) is a complex cellular network that allows an appropriate reaction to different DNA lesions. Depending on the type and severity of DNA lesions, the cellular response ranges from successful repair to the elimination of damaged cells by apoptosis. While repair pathways aim to remove or tolerate damage and preserve cellular functionality, DNA damage-induced apoptosis utilizes cell death as a protective mechanism to prevent cells becoming dysfunctional or malignant [1–3]. This mechanism is particularly relevant in oncological diseases, as cancer cells are characterized by genomic instability and evasion of apoptosis [4]. Dysregulation of DNA damage-induced apoptosis affects carcinogenesis, cancer maintenance, therapy and resistance [5–7]. Thus, genes involved in this pathway are frequently mutated in cancer cells and alterations in apoptosis genes particularly correlate with a lower overall survival compared to alterations in repair genes [8, 9].

Since the majority of oncological diseases are multifactorial, their genetic architecture is defined by various common single nucleotide polymorphisms (SNPs), each with a small effect size [10, 11]. Genome-wide association studies (GWAS) have identified numerous SNPs associated with different cancer entities [12, 13]. As these SNPs are predominantly located in non-coding regions, they affect the regulation of gene expression rather than altering the gene itself [14, 15]. By integrating genotype with expression data, expression quantitative trait loci (eQTL) analyses facilitate the functional characterization of regulatory variants [16–19]. To account for exogenous factors that influence regulatory processes under pathophysiological conditions, the analysis of exposure eQTL (e^2^QTL) allows for the identification of context-specific effects and enables the explanation of previously undetected mechanisms [20–23]. With regard to the ability of damaged cells to evade apoptosis, previous studies have shown that DNA-damaging agents provoke apoptosis by transcriptionally distinct responses and are accompanied by a selective downregulation of genes, including DNA repair genes [24–26]. In addition, many DDR proteins play a dual role in repair and apoptosis, which is mediated by posttranslational modifications [27, 28]. However, it is still unclear how the decision to repair lesions or commit apoptosis is made and how these processes are regulated. Thus, the analysis of e^2^QTL in the context of DNA damage-induced apoptosis is fundamental for understanding pathomechanisms and developing new prevention and treatment strategies.

In this study, we analyze the cellular mechanisms of DNA damage-induced apoptosis with specific interest in how inter-individual genetic variability influences their regulation. Cytotoxic CD8^+^ T cells are chosen because monocytes as well as resting T cells are impaired in DNA repair [29, 30]. We stimulate these cells with high doses of 5 different DNA-damaging agents to cover the broad spectrum of responses to DNA damage-induced apoptosis. Chemical alterations of single bases are induced using the alkylating agent Methyl-methanesulfonate (MMS) [31] and the reactive oxygen species tert-butyl-hydroperoxide (TBOOH) [32]. Furthermore, we utilize benzo(a)pyrene-7,8-diol-9,10-epoxide (BPDE) [33] and ultraviolet (UVC) radiation [34] to cause bulky lesions like adducts or dimers. The preferred repair pathway for the former chemical lesions (MMS, TBOOH) is base excision repair (BER), whereas the latter bulky lesions (BPDE, UVC) predominantly address nucleotide excision repair (NER) [35]. Additionally, we induce a third kind of DNA damage by stimulating cells with 4-hydroxycyclophosphamide (HC) [36]. The resulting DNA crosslinks are repaired by a complex interplay of different pathways, including excision-based repair mechanisms, homologous recombination (HR) and the Fanconi anaemia (FA) pathway [37]. Our results exhibit regulatory mechanisms in the context of DNA damage-induced apoptosis ranging from a general inhibition of transcription to eQTL and e^2^QTL that act in a stimulus specific manner. Finally, we test whether eQTL and e^2^QTL represent risk variants from GWAS for multifactorial diseases and, thus, provide further insights into the pathomechanisms of corresponding phenotypes.

## Methods

### Sample collection, isolation and stimulation of CD8^+^ T cells

The study includes blood samples from 461 healthy European donors (non-smoker, no infection or vaccination 4 weeks prior to blood withdrawal, white blood count (WBC) < 10 × 10^3^/ μl). We isolated peripheral blood mononuclear cells (PBMCs) of all participants by density gradient centrifugation using Pancoll (PAN biotech). CD8^+^ T cells were isolated by magnetic cell separation using CD8 MicroBeads (Milteny Biotec) according to manufacturer’s instructions. Cell purity > 95% was evaluated based on FACS analysis (CytoFLEX, Beckmann Coulter Life Science) with an antibody against CD8 (Milteny Biotec). Cells were cultured in TexMACS GMP medium (Milteny Biotec) supplemented with 50 µg/ml Gentamycin (Gibco) in a density of 2,000,000 cells in 400 μl overnight. Stimulation of cells was performed the next day by dividing cells of each participant into six subsets that were either left untreated or treated with 5 µM benzo(a)pyrene-7,8-diol-9,10-epoxide (Toronto Research Chemicals), 0.5 mM 4-hydroxycyclophosphamide (Cayman Chemical), 0.5 mM tert-butyl-hydroperoxide (Sigma-Aldrich), 1 nM Methyl-methanesulfonate (Sigma-Aldrich) for 6 h or irradiated with 50 mJ/ cm^3^ UVC (Stratalinker 2400, Stratagene). Dose and duration of stimulation were determined based on the highest number of differentially expressed genes (DEGs) (Additional file 1: Table S1). Cell viability and induced apoptosis were assessed by Annexin V/ PI assay (Biozol) and Trypan Blue Exclusion Test (Invitrogen). After treatment cells were lysed using RLT reagent (Qiagen) and stored at -80 °C.

### DNA and RNA isolation

DNA of untreated CD8^+^ T cells and RNA of untreated and all stimulated CD8^+^ T cells were isolated using the AllPrep 96 DNA/RNA Kit from Qiagen. Quantity of nucleic acids was determined using a microplate reader (Varioskan LUX, Thermo Fisher Scientific) and appropriate Quant-iT kits (Invitrogen). Quality was determined by electrophoresis (TapeStation, Agilent Technologies) for a subset of samples.

### Genotyping and imputation

Participants were genotyped using DNA of untreated CD8^+^ T cells. Genotyping was conducted on Infinium Global Screening Array v3.0 (Illumina) and raw data was processed using the GenomeStudio Software (Illumina). Quality control (QC) of generated PLINK [38] files included minor allele frequency (MAF) > 5%, call rate > 97% and Hardy–Weinberg equilibrium (HWE) (P_HWE_ > 10^–4^) as well as statistics for sex, kinship, heterozygosity and population stratification based on 1000 Genomes reference data as recommended [39–41]. A total of 482,620 SNPs were used as input for the TOPMed Imputation Server [42–44].

### Gene expression analysis

RNA of untreated and stimulated CD8^+^ T cells was used for library preparation. Libraries were generated using QuantSeq FWD (Lexogen) with unique dual indices according to manufacturer’s instructions and sequenced on NovaSeq6000 (Illumina) with a read length of 100 bp and 10 million reads per sample. Raw data was processed using BBDuk [45] for adapter trimming and STAR [46] for alignment. QC included FastQC [47], RSeQC [48] and MultiQC [49]. An additional quality control step was added based on expression data by performing principal component (PC) analysis of control samples together with a single stimulus. We used the Gaussian mixture model of the scikit-learn library [50] to predict clusters based on the first two PCs using k-means clustering algorithm for initialization. After removing identified outliers, the final dataset consisted of 2,728 samples and 60,617 genes for further analysis. Differential expression analysis was performed for each stimulus separately as described by Law et al. [51] using edgeR [52] and limma [53]. Multiple testing correction was performed using the Benjamini–Hochberg method [54].

### eQTL and e^2^QTL analysis

We used FastQTL [55] to analyze cis-eQTL within a 1 megabase window of a gene’s transcription start site. Filtering and normalization of expression data was performed as described by the Genotype Tissue Expression (GTEx) project [56] including 60 PEER factors, top 3 genotype PCs and sex as covariates. Genotypes were filtered by P_HWE_ > 10^–6^ and MAF > 5%. Adjusted p-values were generated using 1,000 to 10,000 permutations. Based on our eQTL data, we determined the most significant variant for each analyzed gene (referred to as top eQTL) to calculate e^2^QTL in a z-test and corrected for multiple testing using Bonferroni correction as described by Kim-Hellmuth et al. [23].

### Enrichment

Enrichment of Gene Ontology (GO) terms [57, 58] as well as Gene Set Enrichment Analysis (GSEA) [59, 60] were performed using clusterProfiler [61]. GO terms only included biological processes and GSEA was performed for hallmark gene sets of the Molecular Signatures Database (MSigDB) [62] with all parameters set to default. Categories with a q-value < 0.05 were reported as significantly enriched.

### Trait association and colocalization

Associations of eQTL and genome-wide significant associated GWAS variants were assessed using the Open Targets Platform [63, 64]. GWAS variants had to be a significant eQTL (qval < 0.05) and in strong linkage disequilibrium (LD) (r^2^ > 0.7) to the top eQTL variant to be reported. LD was calculated using LDlink [65]. If GWAS summary statistics were available, colocalization analysis was performed using coloc [23] with all parameters set to default.

## Results

### Study setup and expression profiling of CD8^+^ T cells upon carcinogen treatment

**Fig. 1:**
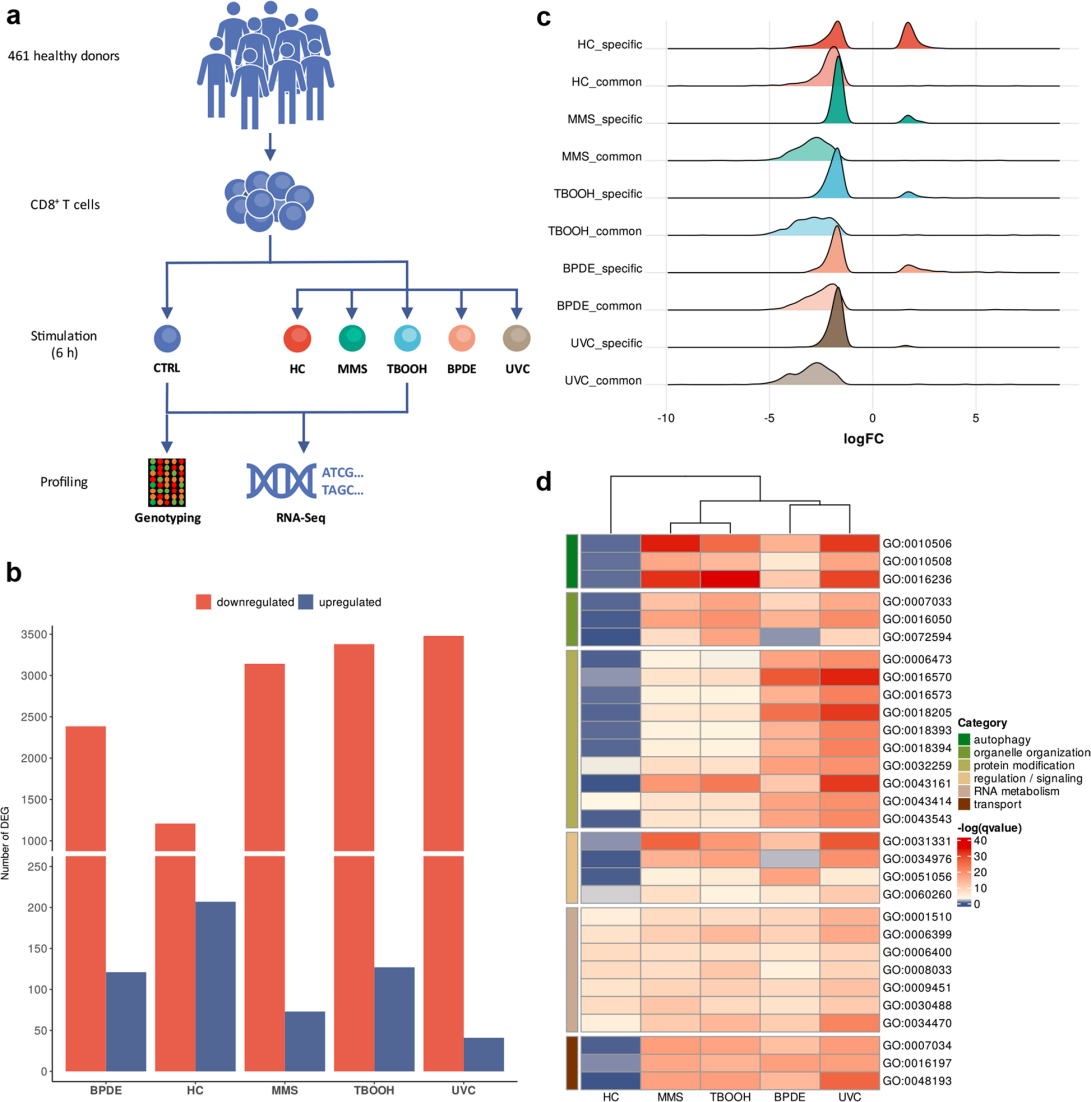
Study setup and expression profiling of CD8^+^ T cells upon carcinogen treatment. **a** Overview of the study design. Blood samples from 461 healthy donors were used to isolate CD8^+^ T cells. Cells were either left untreated or stimulated with different carcinogens for 6 h. Expression analysis and genotype profiling were performed to identify regulatory variants. **b** Number of significantly downregulated (red) and upregulated (blue) genes (adj. *p* < 0.05 and abs(logFC) > 1.5) identified for each stimulus. **c** Distribution of the logFC for significant DEG (adj. *p* < 0.05 and abs(logFC) > 1.5) in each stimulus. Genes were classified as specific (significant only in one stimulus) and common genes (significant in two or more stimuli). **d** Clustered heatmap of significantly enriched GO terms for biological processes of downregulated genes. Hierarchical clustering was performed using complete-linkage clustering and Euclidean distances. BPDE—benzo(a)pyrene-7,8-diol-9,10-epoxide, HC—4-hydroxycyclophosphamide, MMS—Methyl-methanesulfonate, TBOOH—tert-butyl-hydroperoxide, UVC—ultraviolet radiation

To examine regulatory processes of DNA damage-induced apoptosis, we isolated CD8^+^ T cells from blood samples obtained from 461 healthy donors. We stimulated cells for 6 hours (h) using high doses of 5 different DNA-damaging agents to induce early apoptosis. As the DDR is specific to different types of lesions, we selected MMS, TBOOH, UVC, BPDE, and HC to address a broad spectrum of responses. Transcriptional analysis was performed of all stimulated cells as well as untreated controls by RNA-Sequencing. In addition, all donors were genotyped genome-wide by analyzing the untreated controls on Global Screening Array v3.0 (Fig. 1a).

Expression profiling revealed a total of 5,373 differentially expressed genes (DEG) across all stimuli (adj. p < 0.05 and abs(logFC) > 1.5) (Additional file 1: Table S2). We found a high number of repressed genes in all conditions ranging from 85% in HC to 99% in UVC (Fig. 1b, Additional File 2: Figure S1a). To exclude the observed repression is due to RNA degradation, we performed extensive quality control steps (see Methods). In addition, we evaluated the count distribution for *CD8A*, *CD8B* and the reference genes *GAPDH* and *UBE2D2* and did not find a general reduction in the expression of these genes upon stimulation (Additional file 2: Figure S1b). These results were in line with differential expression analysis showing no significant downregulation of the genes (logFC > -1.5). Our results were consistent with prior research indicating an inhibition of transcription and induced apoptosis upon exposure to high dose DNA-damaging agents [26]. Notably, data of a previous study by Lu et al. [25] analyzing expression response of epithelial cells to high dose 0.5 μM BPDE matched our results regarding the direction of fold change (FC). Based on our significant DEG we found 414 common genes in both datasets with 99% indicating the same FC direction (Additional File 1: Table S3).

To analyze expression patterns with respect to regulatory differences across stimuli, we categorized DEG into common genes, that were significant across all stimuli, and specific genes, that were significant in only one stimulus. Common DEG were predominantly repressed, while specific DEG exhibited induction or repression (Fig. 1c). Given the general inhibition of transcription, we conducted a grouped enrichment analysis of Gene Ontology (GO) terms for biological processes [57, 58] using all significantly downregulated genes (Additional file 1: Tab S4). To evaluate stimulus-specific differences, we determined the 10 most significant terms per stimulus and performed hierarchical cluster analysis. Subclusters were identified for stimuli inducing chemical alterations (TBOOH and MMS) or bulky lesions (BPDE and UVC). While genes downregulated upon treatment with TBOOH or MMS showed enrichment for terms associated with autophagy, organelle organization or transport, genes downregulated upon treatment with BPDE or UVC predominantly showed enrichment for terms associated with protein modifications. Of note, genes downregulated upon treatment with HC only exhibited significant enrichment for GO terms related to RNA metabolism (Fig. 1d, Additional file 1: Table S5).

Due to the small number of upregulated genes and their tendency to be stimulus specific, only 8 common induced genes were identified, including the spliceosomal RNA *U2* and the snRNAs *RNU4-2* and *SNORD3A* (Additional file 2: Figure S1c). Gene set enrichment analysis (GSEA) [59, 60] for all induced genes determined apoptosis (q = 1.22e^−4^), p53 signaling (q = 1.22e^−4^), oxidative phosphorylation (q = 2.17e^−2^) and E2F targets (q = 4.6e^−2^) as significant hallmarks. However, it should be noted that these results mainly rely on genes induced by BPDE and HC, because stimulation with all other exposures exhibited no significant enrichment due to the smaller number of upregulated genes (Additional file 1: Table S6, Additional file 2: Figure S1d).

### Identification of eQTL and e^2^QTL in the context of DNA damage-induced apoptosis

In order to assess how genetic variability influences DNA damage-induced apoptosis based on gene expression, we performed eQTL and e^2^QTL analyses. Here, we focused on cis-eQTL that were defined as eQTL within a 1 MB window of a gene’s transcription start site. All eQTL were calculated using FastQTL [55] and e^2^QTL were defined by comparing the regression slope of the most significant variant for each analyzed gene (referred to as top eQTL) in unstimulated and stimulated cells in a z-test as described in Kim-Hellmuth et al. [23].

**Fig. 2:**
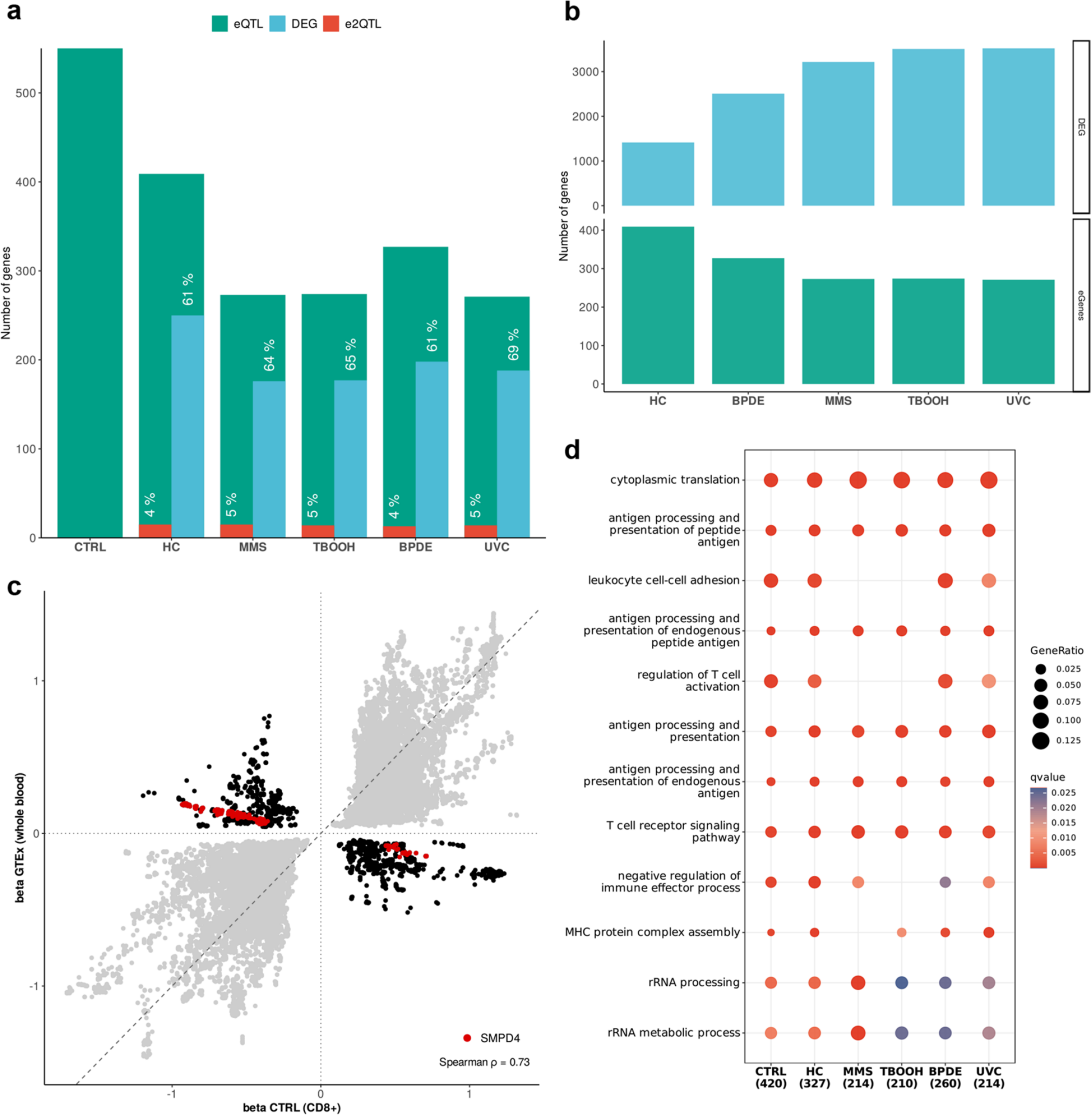
Identification of eQTL and e^2^QTL in the context of DNA damage-induced apoptosis. **a** Number of eGenes with at least one significant eQTL separated by stimulus. The fraction of eGenes that are significant DEGs (blue) or significant e^2^QTL (red) is indicated. eQTL under unstimulated condition are referred to as control (CTRL). **b** Comparison of the number of significant DEGs (blue) and eGenes (green) for each stimulus. **c** Scatter plot of eQTL effect size beta for eQTL present in both GTEx whole blood and our untreated CD8^+^ T cell data (referred to as CTRL). eQTL with identical direction of effect size are shown in grey and eQTL with opposite effect size are shown in black. eQTL of *SMPD4* are highlighted in red. **d** Enrichment analysis of eGenes for GO terms associated with biological processes separated by stimulus. The gene ratio is indicated by the size and the q-value by the color of dots. Numbers under each stimulus indicate the number of eGenes included in the analysis. eGenes under unstimulated condition are referred to as control (CTRL). BPDE—benzo(a)pyrene-7,8-diol-9,10-epoxide, HC—4-hydroxycyclophosphamide, MMS—Methyl-methanesulfonate, TBOOH—tert-butyl-hydroperoxide, UVC—ultraviolet radiation

Across all conditions, we found 654 genes (called eGenes) that were significantly regulated by eQTL (qval < 0.05) (Additional file 1: Table S7). Of these, 49% were specific for one stimulus and 20% were shared across all stimuli. Especially cells treated with HC showed a high fraction of stimulus specific eGenes (Additional file 2: Figure S2a). For 47 of all identified eGenes, we found additional e^2^QTL effects due to differences in genetic regulation upon stimulation (Additional file 1: Table S8), which represented a fraction of 4% to 5% of eGenes per stimulus (Fig. 2a). In order to evaluate our eQTL results with regard to DEG, 61% to 69% of eGenes were differentially expressed including the identified e^2^QTL. Noteworthy, 550 of 654 identified eGenes were found in unstimulated CD8^+^ T cells, with smaller numbers reaching from 409 to 271 eGenes for stimulated T cells. To understand why the number of eQTL dropped upon stimulation, we compared effect sizes of eQTL before and after stimulation and found a reduction of slopes in > 60% of eQTL in all stimuli (Additional file 2: Figure S2b). In addition, we observed an inverse correlation of eGenes and DEG (Fig. 2b) indicating a reduced genetic effect on gene regulation upon stimulation. This tendency was previously described in a single cell eQTL study of immune cells [66].

As we used CD8^+^ T cells for our study, we were interested in the cell type specificity of the identified eGenes. Therefore, we compared the effect sizes of our eQTL under basis condition to whole blood eQTL data of the Genotype Tissue Expression project (GTEx) [56]. By analyzing all eQTL present in both datasets the Spearman coefficient for the correlation of effect sizes was 0.73. While the direction of the regression slopes was identical for most of the eQTL, we found 38 eGenes with a consistently opposite slope in GTEx and our data (Fig. 2c, Additional file 1: Table S9). Of these, *SMPD4* (*NSMASE3*) was reported as e^2^QTL in our study with an absolute difference of slopes to GTEx reaching from 0.44 to 1.12. Furthermore, we conducted an enrichment analysis of GO terms for biological processes using all eGenes. We found leukocyte and T cell specific processes to be enriched but also observed stimulus specific differences. While cells treated with HC exhibited the highest number of eGenes upon stimulation, the enrichment of GO terms was more similar to untreated cells compared to other conditions. Moreover, stimuli addressing BER (MMS, TBOOH) likewise lacked enrichment of single leukocyte specific processes that were still significant enriched for stimuli addressing NER (BPDE, UVC) (Fig. 2d, Additional file 1: Table S10).

### Trait-association of eQTL and e^2^QTL based on GWAS data

Since our results have already illustrated regulatory characteristics of DNA damage-induced apoptosis, we completed our analyses by evaluating the role of eQTL and e^2^QTL variants for multifactorial diseases. We utilized the Open Targets Platform [63, 64] and tested whether risk variants that have been identified by GWAS for multifactorial diseases represent top eQTL or e^2^QTL. Here, we filtered for genome-wide significant associated GWAS variants that represent significant eQTL or e^2^QTL in our study (qval < 0.05) and are in strong linkage disequilibrium (LD, *r*^2^ > 0.7) to the top eQTL or e^2^QTL. In addition to all significant e^2^QTL, we only included eQTL with a strong differential expression (adj. *p* < 0.05 and abs(logFC) > 2) to ensure that the eQTL effects are in context with the stimulation and induced DDR.

Overall, we identified 3 eQTL and 8 e^2^QTL referring to 7 different eGenes that represent GWAS risk variants for 5 oncological and 2 neuropsychiatric diseases (Table 1).

**Table 1:** eQTL and e^2^QTL that represent GWAS risk variants for multifactorial diseases

**Gene**	**Stimulus**	**Trait**	**GWAS variant**	**eQTL / e**^**2**^**QTL variant**	**beta**	**q-value**	**LD *r***^**2**^
*XBP1*	TBOOH	Breast Cancer	rs5997389 (G/**A**)	rs3788408 (A/**G**)	0.51	4.77E-04	0.802
	TBOOH	Ovarian Cancer	rs6005807 (T/**C**)	rs3788408 (A/**G**)	0.51	4.77E-04	0.73
	HC	Ovarian Cancer	rs6005807 (T/**C**)	chr22:28639216:A:ATAAG*	0.54	1.37E-06	0.746
*PLEC*	BPDE	Bipolar Disorder	rs6992333 (A/**G**)	rs7822511 (T/**C**)	-0.35	1.77E-04	0.852
		Multiple Sclerosis	rs3923387 (C/**T**)	rs7822511 (T/**C**)	-0.35	1.77E-04	0.804
*KLF2*	MMS	Multiple Myeloma	rs11086029 (**T**/A)	rs3745318 (T/**C**)	-0.42	6.94E-15	0.833
	BPDE	Multiple Myeloma	rs11086029 (**T**/A)	rs2362475 (A/**C**)	-0.43	5.53E-18	0.983
	UVC	Multiple Myeloma	rs11086029 (**T**/A)	rs7246538 (A/**G**)	-0.43	9.00E-15	0.907
	TBOOH	Multiple Myeloma	rs11086029 (**T**/A)	rs4808046 (G/**A**)	-0.39	7.94E-19	0.983
*PIP4K2A*	MMS	B cell ALL	rs2296624 (**C**/T)	rs10430590 (A/**T**)	-0.32	1.36E-03	0.761
*GPR160*	TBOOH	Testicular germ cell tumor	rs3755605 (C/**T**)	rs6778888 (C/**T**)	0.39	3.47E-02	0.824
*RPS18*	HC	Breast Cancer	rs17215231 (C/**T**)	rs17215231 (C/**T**)	-0.80	6.86E-10	1
*ARL17B*	UVC	Breast Cancer	rs199533 (**G**/A)	rs141783865 (A/**C**)	0.84	2.60E-27	0.91

*BPDE* benzo(a)pyrene-7,8-diol-9,10-epoxide, *HC – 4* hydroxycyclophosphamide, *MMS* Methyl-methanesulfonate, *TBOOH* tert-butyl-hydroperoxide, *UVC* ultraviolet radiation, *ALL* acute lymphoblastic leukaemia

Effects of *XBP1* and *PLEC* refer to eQTL (upper part of the table) and effects of *KLF2*, *PIP4K2A*, *GPR160*, *RPS18* and *ARL17B* refer to e^2^QTL (lower part of the table). Effect alleles are highlighted in bold. Variants are indicated by an asterisk (*) if rs ID is not available. Effect size beta and q-value refer to the eQTL effect upon stimulation or e^2^QTL effect, respectively

**Fig. 3:**
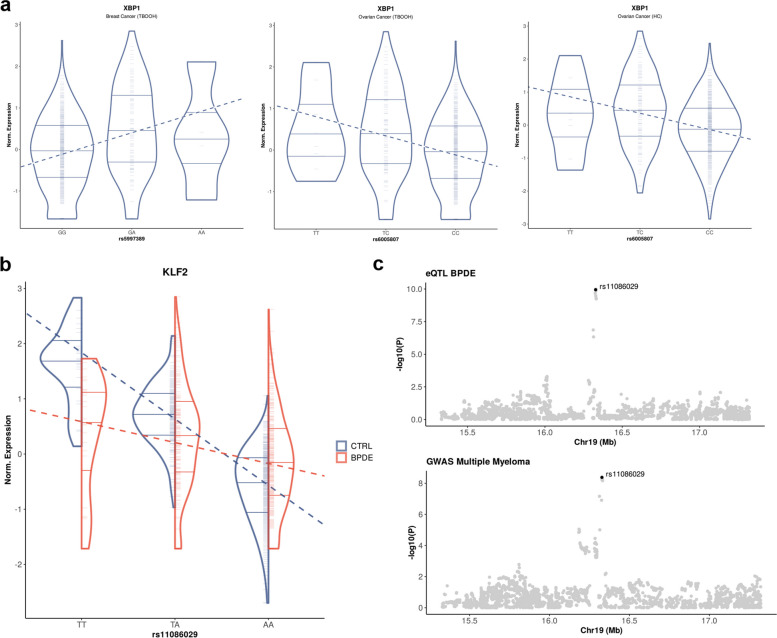
Trait association of eQTL and e^2^QTL based on GWAS data. **a** Violin plots of the eQTL for XBP1 in breast cancer upon TBOOH treatment (1), ovarian cancer upon TBOOH treatment (2) and ovarian cancer upon HC treatment (3). Genomic variants on the x-axis represent GWAS lead variants for the corresponding trait. Individuals are indicated by small ticks and horizontal lines represent the median as well as the 25% and 75% quartiles in each violin. The dashed lines indicate the linear regression for the effect of the genotype on gene expression. **b** Violin plots of the e^2^QTL for KLF2 in untreated cells (CTRL, blue) and upon BPDE treatment (red). The genomic variant on the x-axis represents the GWAS lead variant for MM. **c** Regional association plots for common variants in our eQTL data upon BPDE treatment and the GWAS for MM. The lead variant of the MM GWAS is highlighted in black and annotated, respectively. BPDE—benzo(a)pyrene-7,8-diol-9,10-epoxide, HC—4-hydroxycyclophosphamide, MM – Multiple Myeloma, TBOOH—tert-butyl-hydroperoxide

Of the disease related eQTL (Additional file 1: Table S11), both eGenes represent significant downregulated DEG and their disease association is specific to distinct stimuli. The top eQTL for *PLEC* upon BPDE exposure is in strong LD with GWAS risk variants for bipolar disorder [67] (*r*^2^ = 0.852) and multiple sclerosis [68] (*r*^2^ = 0.804). Carriers of the GWAS risk alleles exhibit decreased expression of *PLEC* in both traits (Table 1). The top eQTL for *XBP1* represents a GWAS risk variant for breast cancer [69] upon TBOOH treatment (*r*^2^ = 0.802) and for ovarian cancer [70] upon TBOOH treatment (*r*^2^ = 0.73) and HC treatment (*r*^2^ = 0.746). The GWAS risk allele associated with breast cancer leads to an increased *XBP1* expression, while the opposite effect is observed for the GWAS risk alleles associated with ovarian cancer (Table 1, Fig. 3a).

Of all GWAS related e^2^QTL (Additional file 1: Table S12), only *KLF2* represents a significant downregulated DEG and the disease associations are present across all BER and NER associated stimuli, whereas all other eGenes show not differential expression but are stimulus specific. We found that the e^2^QTL for *ARL17B* upon UVC and *RPS18* upon HC exposure represent GWAS risk variants for breast cancer [71, 72] (*r*^2^ = 0.91, *r*^2^ = 1), both exhibiting reduced expression in carriers of the risk alleles (Table 1). Of note, *RPS18* is the only eGene exhibiting a stronger eQTL effect upon stimulation and the same variant for the GWAS and the top e^2^QTL (Additional file 2: Figure S3a). Furthermore, the top e^2^QTL for *GPR160* upon TBOOH exposure represents a GWAS risk variant for testicular germ cell tumors [73] (*r*^2^ = 0.824) and the e^2^QTL for *PIP4K2A* upon MMS exposure represents a GWAS risk variant for B cell acute lymphoblastic leukaemia (ALL) [74] (*r*^2^ = 0.761). Both findings exhibit increased gene expression for carriers of the GWAS risk alleles and reduced eQTL effect sizes upon stimulation (Table 1).

We were particularly interested in the e^2^QTL for *KLF2* that represent a GWAS risk variant for multiple myeloma (MM) [75] and are present among all stimuli associated to BER and NER (*r*^2^ = 0.833 – 0.983). The e^2^QTL for *KLF2* exhibit the strongest difference of slopes before and after treatment in our analysis (abs(∆_slope_) = 0.7 – 0.75). For all conditions we observed an increased expression of *KLF2* in carriers of the GWAS risk alleles and reduced eQTL effect sizes upon stimulation that matches the observed DEG effect (Table 1, Fig. 3b, Additional file 2: Figure S3b). As summary statistics for the MM GWAS were available, we additionally tested whether the GWAS and our e^2^QTL data are likely to share a causal variant at the *KLF2* locus using coloc [76]. We found PP.H4 > 0.993 and PP.H4/PP.H3 > 143 in all conditions confirming strong evidence of sharing the same causal variant in our e^2^QTL analysis and the MM GWAS (Fig. 3c, Additional file 2: Figure S3b).

Altogether, our results demonstrate the relevance of regulatory variants in the context of DNA damage-induced apoptosis and highlight their role in oncological diseases.

## Discussion

In this study, we analyzed regulatory mechanisms in the context of DNA damage-induced apoptosis with a particular interest in eQTL and e^2^QTL effects and their role for multifactorial diseases. Even though genomic instability and evading apoptosis are known hallmarks for various diseases, less is known about the inter-individual genetic influence on the underlying biological processes. Our results offer new insights into the regulation of DNA damage-induced apoptosis as well as provide genes and cellular mechanisms that might be relevant for future research.

Consistent with previous studies, differential expression analysis revealed a general inhibition of transcription with no upregulation of specific repair pathways. Our results further support the assumption that apoptosis induced by high dose DNA-damaging agents does not lead to a general shutdown of cellular processes but is accompanied by a selective downregulation of genes [24, 25]. Due to the high number of suppressed genes, we had a particular interest in the upregulated genes. Except for the spliceosomal RNA *U2*, the majority of commonly induced genes is poorly characterized, but at least for the snRNAs *RNU4-2* and *SNORD3A*, cancer-related functionalities were reported previously [77–79]. Especially *SNORD3A* differs from other upregulated genes by a conspicuous strong induction (logFC > 6) across all conditions, emphasizing the role of regulatory RNAs in the context of DNA damage-induced apoptosis. Moreover, other genes upregulated in distinct but also specific stimuli like *PPA1* [80, 81], *GADD45B* [82], *C1D* [83], *TRPV2* [84] and *RPS14* [85, 86] were associated with DDR, cancer and apoptosis. Therefore, our results reflect known characteristics of DNA damage-induced apoptosis and underline their relevance in oncological disease but also highlight the importance of still unexplained genes and regulatory networks.

To evaluate how inter-individual genetic variability influences DNA damage-induced apoptosis, we analyzed eQTL and e^2^QTL. On the one hand, smaller effect sizes of eQTL upon stimulation as well as the inverse correlation of eGenes and DEG support earlier hypotheses of a restricted influence of gene regulatory variants in the cellular response to external stimuli [66]. On the other hand, cell type and context specificity enable to find new and divergent gene regulatory mechanisms, as seen for *SMPD4*, that exhibited opposite eQTL compared to the results of the GTEx project [56] and showed an additional e^2^QTL effect in our study. *SMPD4* is involved in DNA damage-induced apoptosis and dysregulated in several tumors [87], but neither cell type nor context specific expression effects have been described before. Our findings, thus, highlight the specificity of gene regulatory variants in certain biological processes and the need for context specific analyses to explain gene regulatory mechanisms with regard to exogenous factors.

Another goal of our study was to identify eGenes that are associated to multifactorial diseases in order to gain new insights into cellular pathomechanisms. As expected, the majority of associated diseases were oncological and only one eGene was found for neuropsychiatric diseases. Although DDR and apoptosis have already been described as risk factors for multiple sclerosis and bipolar disorder [88, 89], the associated gene *PLEC* has not been described in the context of these diseases before. *PLEC* encodes a cytolinker protein that is relevant for the structural scaffold as well as signaling of cells, including astrocytes, that are known to play an important role in both traits [90–92]. Of the cancer related eGenes, *XBP1* is relevant for different processes in tumors due to its role in unfolded protein response (UPR). Moreover, its responsiveness to estrogen highlights the importance in estrogen-dependent cancers like breast and ovarian cancer [93]. In line with our results, carriers of the GWAS risk alleles exhibit an increased expression in triple-negative breast cancer (TNBC) cells [94], while ovarian cancer cells show a decreased *XBP1* expression [95]. In addition, context specific regulatory effects for *ARL17B* and *RPS18* were found to be associated with breast cancer. The cellular function of both genes is poorly understood. *RPS18* was not differentially expressed (p.adj = 0.99), but its strong genotype dependent regulation upon stimulation should be considered regarding its usage as housekeeper under certain conditions [96]. Moreover, we found an increased expression of *GPR160* and *PIP4K2A* among carriers of GWAS risk variants for testicular germ cell tumors and B cell ALL. These results further support previous studies reporting high expression of *GPR160* and *PIP4K2A* in corresponding cancer cells that undergo apoptosis after knockdown of the corresponding genes in both cases [97, 98]. Together, these data indicate that DNA damaged cells from carriers of *GPR160* and *PIP4K2A* risk alleles might be at higher risk to evade apoptosis. Thus, both context specific targets may help to improve therapy and overcome resistances of both cancer entities in future. At least, we found that regulatory effects of *KLF2* are related to MM across BER and NER associated stimuli. *KLF2* is a transcription factor involved in various biological processes and is discussed as tumor suppressor for several cancer entities [99, 100]. MM cells are characterized by an increased *KLF2* expression and knockdown of *KLF2* leads to apoptosis, which further highlights the relevance of *KLF2* in MM maintenance [101]. The findings are in line with our e^2^QTL effects which show that carriers of the MM risk allele exhibit a stronger *KLF2* downregulation than carriers of the opposite allele. Furthermore, the fact that all conditions except from HC showed e^2^QTL effects, indicates that single base modifications that induce BER and NER are more likely to contribute to *KLF2* related MM pathophysiology.

Despite the promising results of this analysis, our study has limitations. We used CD8^+^ T cells as model cell type to study the influence of genetic variability on DNA damage-induced apoptosis. However, we cannot determine to what extent our data on apoptosis can be transferred to other cell types. In addition, all studies were performed in European individuals. It is, thus, unclear to what extent the results can be transferred to non-European populations.

## Conclusions

This analysis provides new insights into the context specific regulation of gene expression by genetic variants. As evading apoptosis is a key hallmark of cancer, particularly the regulation of DNA damage-induced apoptosis is crucial to understand the pathophysiology of oncological diseases. Our study reveals individual regulatory mechanisms and corresponding genes that promote apoptosis. The findings might represent promising targets for future research in the field of apoptosis related diseases.

## Supplementary Information

Additional file 1: Master file for Tables S1-S12. (Excel https://www.microsoft.com/de-de/microsoft-365/excel?market=de).

Additional file 2: Supplementary figures. (pdf, Acrobat Reader https://www.adobe.com/de/acrobat.html).

## Data Availability

The raw data of this study are available from Inventum Genetics GmbH but restrictions apply to the availability of these data, which were used under license for the current study. Data are however available from the authors upon reasonable request and with permission of Inventum Genetics GmbH. Data has been deposited at the European Genome-phenome Archive (EGA) under accession number EGAS50000000666. Processed data that support the findings is either included in the published article and its supplementary information files or publicly available at the Gene Ontology knowledgebase [57, 58], the Genotype-Tissue Expression project [56], the Molecular Signatures Database [62] or the Open Targets Platform [63, 64].

## Abbreviations

ALL: Acute lymphoblastic leukaemia
BER: Base excision repair
BPDE: Benzo(a)pyrene-7,8-diol-9,10-epoxide
DDR: DNA damage response
DEG: Differentially expressed genes
e^2^QTL: Exposure expression quantitative trait loci
eQTL: Expression quantitative trait loci
FA: Fanconi anaemia
FC: Fold change
GO: Gene Ontology
GSEA: Gene Set Enrichment Analysis
GTEx: Genotype Tissue Expression
GWAS: Genome-wide association study
HC: 4-Hydroxycyclophosphamide
HR: Homologous recombination
HWE: Hardy-Weinberg equilibrium
LD: Linkage disequilibrium
MAF: Minor allele frequency
MM: Multiple Myeloma
MMS: Methyl-methanesulfonate
MSigDB: Molecular Signature DataBase
NER: Nucleotide excision repair
PBMC: Peripheral blood mononuclear cells
PC: Principal component
QC: Quality control
SNP: Single nucleotide polymorphism
TBOOH: Tert-butyl-hydroperoxide
TNBC: Triple negative breast cancer
UPR: Unfolded protein response
UVC: Ultraviolet radiation
WBC: White blood count

## References

[1] Harper JW, Elledge SJ. The DNA damage response: ten years after. Mol Cell. 2007;28:739–45. 10.1016/j.molcel.2007.11.015. 18082599 10.1016/j.molcel.2007.11.015

[2] Jackson SP, Bartek J. The DNA-damage response in human biology and disease. Nature. 2009;461:1071–8. 10.1038/nature08467. 19847258 10.1038/nature08467 PMC2906700

[3] Ciccia A, Elledge SJ. The DNA damage response: making it safe to play with knives. Mol Cell. 2010;40:179–204. 10.1016/j.molcel.2010.09.019. 20965415 10.1016/j.molcel.2010.09.019 PMC2988877

[4] Hanahan D, Weinberg RA. Hallmarks of cancer: the next generation. Cell. 2011;144:646–74. 10.1016/j.cell.2011.02.013. 21376230 10.1016/j.cell.2011.02.013

[5] Bouwman P, Jonkers J. The effects of deregulated DNA damage signalling on cancer chemotherapy response and resistance. Nat Rev Cancer. 2012;12:587–98. 10.1038/nrc3342. 22918414 10.1038/nrc3342

[6] O’Connor MJ. Targeting the DNA damage response in cancer. Mol Cell. 2015;60:547–60. 10.1016/j.molcel.2015.10.040. 26590714 10.1016/j.molcel.2015.10.040

[7] Bartkova J, Horejsí Z, Koed K, Krämer A, Tort F, Zieger K, et al. DNA damage response as a candidate anti-cancer barrier in early human tumorigenesis. Nature. 2005;434:864–70. 10.1038/nature03482. 15829956 10.1038/nature03482

[8] Knijnenburg TA, Wang L, Zimmermann MT, Chambwe N, Gao GF, Cherniack AD, et al. Genomic and molecular landscape of DNA damage repair deficiency across the cancer genome atlas. Cell Rep. 2018;23:239–254.e6. 10.1016/j.celrep.2018.03.076. 29617664 10.1016/j.celrep.2018.03.076 PMC5961503

[9] Auslander N, Wolf YI, Koonin EV. Interplay between DNA damage repair and apoptosis shapes cancer evolution through aneuploidy and microsatellite instability. Nat Commun. 2020;11:1234. 10.1038/s41467-020-15094-2. 32144251 10.1038/s41467-020-15094-2 PMC7060240

[10] Manolio TA, Collins FS, Cox NJ, Goldstein DB, Hindorff LA, Hunter DJ, et al. Finding the missing heritability of complex diseases. Nature. 2009;461:747–53. 10.1038/nature08494. 19812666 10.1038/nature08494 PMC2831613

[11] Hindorff LA, Gillanders EM, Manolio TA. Genetic architecture of cancer and other complex diseases: lessons learned and future directions. Carcinogenesis. 2011;32:945–54. 10.1093/carcin/bgr056. 21459759 10.1093/carcin/bgr056 PMC3140138

[12] Sud A, Kinnersley B, Houlston RS. Genome-wide association studies of cancer: current insights and future perspectives. Nat Rev Cancer. 2017;17:692–704. 10.1038/nrc.2017.82. 29026206 10.1038/nrc.2017.82

[13] Sato G, Shirai Y, Namba S, Edahiro R, Sonehara K, Hata T, et al. Pan-cancer and cross-population genome-wide association studies dissect shared genetic backgrounds underlying carcinogenesis. Nat Commun. 2023;14:3671. 10.1038/s41467-023-39136-7. 37340002 10.1038/s41467-023-39136-7 PMC10282036

[14] Maurano MT, Humbert R, Rynes E, Thurman RE, Haugen E, Wang H, et al. Systematic localization of common disease-associated variation in regulatory DNA. Science. 2012;337:1190–5. 10.1126/science.1222794. 22955828 10.1126/science.1222794 PMC3771521

[15] Visscher PM, Wray NR, Zhang Q, Sklar P, McCarthy MI, Brown MA, Yang J. 10 Years of gwas discovery: biology, function, and translation. Am J Hum Genet. 2017;101:5–22. 10.1016/j.ajhg.2017.06.005. 28686856 10.1016/j.ajhg.2017.06.005 PMC5501872

[16] Brem RB, Yvert G, Clinton R, Kruglyak L. Genetic dissection of transcriptional regulation in budding yeast. Science. 2002;296:752–5. 10.1126/science.1069516. 11923494 10.1126/science.1069516

[17] Cheung VG, Spielman RS. Genetics of human gene expression: mapping DNA variants that influence gene expression. Nat Rev Genet. 2009;10:595–604. 10.1038/nrg2630. 19636342 10.1038/nrg2630 PMC2989458

[18] Kim S, Becker J, Bechheim M, Kaiser V, Noursadeghi M, Fricker N, et al. Characterizing the genetic basis of innate immune response in TLR4-activated human monocytes. Nat Commun. 2014;5:5236. 10.1038/ncomms6236. 25327457 10.1038/ncomms6236

[19] Heinrichs SKM, Hess T, Becker J, Hamann L, Vashist YK, Butterbach K, et al. Evidence for PTGER4, PSCA, and MBOAT7 as risk genes for gastric cancer on the genome and transcriptome level. Cancer Med. 2018;7:5057–65. 10.1002/cam4.1719. 30191681 10.1002/cam4.1719 PMC6198243

[20] Abdellaoui A, Yengo L, Verweij KJH, Visscher PM. 15 years of GWAS discovery: realizing the promise. Am J Hum Genet. 2023;110:179–94. 10.1016/j.ajhg.2022.12.011. 36634672 10.1016/j.ajhg.2022.12.011 PMC9943775

[21] Zhang J, Zhao H. eQTL studies: from bulk tissues to single cells. J Genet Genomics. 2023;50:925–33. 10.1016/j.jgg.2023.05.003. 37207929 10.1016/j.jgg.2023.05.003 PMC10656365

[22] Fairfax BP, Humburg P, Makino S, Naranbhai V, Wong D, Lau E, et al. Innate immune activity conditions the effect of regulatory variants upon monocyte gene expression. Science. 2014;343:1246949. 10.1126/science.1246949. 24604202 10.1126/science.1246949 PMC4064786

[23] Kim-Hellmuth S, Bechheim M, Pütz B, Mohammadi P, Nédélec Y, Giangreco N, et al. Genetic regulatory effects modified by immune activation contribute to autoimmune disease associations. Nat Commun. 2017;8:266. 10.1038/s41467-017-00366-1. 28814792 10.1038/s41467-017-00366-1 PMC5559603

[24] Gentile M, Latonen L, Laiho M. Cell cycle arrest and apoptosis provoked by UV radiation-induced DNA damage are transcriptionally highly divergent responses. Nucleic Acids Res. 2003;31:4779–90. 10.1093/nar/gkg675. 12907719 10.1093/nar/gkg675 PMC169943

[25] Lu X, Shao J, Li H, Yu Y. Early whole-genome transcriptional response induced by benzoapyrene diol epoxide in a normal human cell line. Genomics. 2009;93:332–42. 10.1016/j.ygeno.2008.12.007. 19150397 10.1016/j.ygeno.2008.12.007

[26] Ljungman M, Zhang F. Blockage of RNA polymerase as a possible trigger for u.v. light-induced apoptosis. Oncogene. 1996;13:823–31. 8761304

[27] Bernstein C, Bernstein H, Payne CM, Garewal H. DNA repair/pro-apoptotic dual-role proteins in five major DNA repair pathways: fail-safe protection against carcinogenesis. Mutat Res. 2002;511:145–78. 10.1016/s1383-5742(02)00009-1. 12052432 10.1016/s1383-5742(02)00009-1

[28] de Zio D, Cianfanelli V, Cecconi F. New insights into the link between DNA damage and apoptosis. Antioxid Redox Signal. 2013;19:559–71. 10.1089/ars.2012.4938. 23025416 10.1089/ars.2012.4938 PMC3717195

[29] Bauer M, Goldstein M, Christmann M, Becker H, Heylmann D, Kaina B. Human monocytes are severely impaired in base and DNA double-strand break repair that renders them vulnerable to oxidative stress. Proc Natl Acad Sci U S A. 2011;108:21105–10. 10.1073/pnas.1111919109. 22160723 10.1073/pnas.1111919109 PMC3248544

[30] Hu Q, Xie Y, Ge Y, Nie X, Tao J, Zhao Y. Resting T cells are hypersensitive to DNA damage due to defective DNA repair pathway. Cell Death Dis. 2018;9:662. 10.1038/s41419-018-0649-z. 29855463 10.1038/s41419-018-0649-z PMC5981309

[31] Lundin C, North M, Erixon K, Walters K, Jenssen D, Goldman ASH, Helleday T. Methyl methanesulfonate (MMS) produces heat-labile DNA damage but no detectable in vivo DNA double-strand breaks. Nucleic Acids Res. 2005;33:3799–811. 10.1093/nar/gki681. 16009812 10.1093/nar/gki681 PMC1174933

[32] Altman SA, Zastawny TH, Randers L, Lin Z, Lumpkin JA, Remacle J, et al. tert.-butyl hydroperoxide-mediated DNA base damage in cultured mammalian cells. Mutat Res. 1994;306:35–44. 10.1016/0027-5107(94)90165-1. 7512201 10.1016/0027-5107(94)90165-1

[33] Baird WM, Hooven LA, Mahadevan B. Carcinogenic polycyclic aromatic hydrocarbon-DNA adducts and mechanism of action. Environ Mol Mutagen. 2005;45:106–14. 10.1002/em.20095. 15688365 10.1002/em.20095

[34] Rastogi RP, Richa N, Kumar A, Tyagi MB, Sinha RP. Molecular mechanisms of ultraviolet radiation-induced DNA damage and repair. J Nucleic Acids. 2010;2010:592980. 10.4061/2010/592980. 21209706 10.4061/2010/592980 PMC3010660

[35] Lindahl T, Wood RD. Quality control by DNA repair. Science. 1999;286:1897–905. 10.1126/science.286.5446.1897. 10583946 10.1126/science.286.5446.1897

[36] Hengstler JG, Hengst A, Fuchs J, Tanner B, Pohl J, Oesch F. Induction of DNA crosslinks and DNA strand lesions by cyclophosphamide after activation by cytochrome P450 2B1. Mutat Res. 1997;373:215–23. 10.1016/S0027-5107(96)00200-X. 9042403 10.1016/s0027-5107(96)00200-x

[37] Hashimoto S, Anai H, Hanada K. Mechanisms of interstrand DNA crosslink repair and human disorders. Genes Environ. 2016;38:9. 10.1186/s41021-016-0037-9. 27350828 10.1186/s41021-016-0037-9 PMC4918140

[38] Purcell S, Neale B, Todd-Brown K, Thomas L, Ferreira MAR, Bender D, et al. PLINK: a tool set for whole-genome association and population-based linkage analyses. Am J Hum Genet. 2007;81:559–75. 10.1086/519795. 17701901 10.1086/519795 PMC1950838

[39] Auton A, Brooks LD, Durbin RM, Garrison EP, Kang HM, Korbel JO, et al. A global reference for human genetic variation. Nature. 2015;526:68–74. 10.1038/nature15393. 26432245 10.1038/nature15393 PMC4750478

[40] Marees AT, de Kluiver H, Stringer S, Vorspan F, Curis E, Marie-Claire C, Derks EM. A tutorial on conducting genome-wide association studies: Quality control and statistical analysis. Int J Methods Psychiatr Res. 2018;27:e1608. 10.1002/mpr.1608. 29484742 10.1002/mpr.1608 PMC6001694

[41] Turner S, Armstrong LL, Bradford Y, Carlson CS, Crawford DC, Crenshaw AT, et al. Quality control procedures for genome wide association studies. Curr Protoc Hum Genet. 2011;CHAPTER:Unit1.19. 10.1002/0471142905.hg0119s68. 10.1002/0471142905.hg0119s68 PMC3066182 21234875

[42] Das S, Forer L, Schönherr S, Sidore C, Locke AE, Kwong A, et al. Next-generation genotype imputation service and methods. Nat Genet. 2016;48:1284–7. 10.1038/ng.3656. 27571263 10.1038/ng.3656 PMC5157836

[43] Fuchsberger C, Abecasis GR, Hinds DA. minimac2: faster genotype imputation. Bioinformatics. 2015;31:782–4. 10.1093/bioinformatics/btu704. 25338720 10.1093/bioinformatics/btu704 PMC4341061

[44] Taliun D, Harris DN, Kessler MD, Carlson J, Szpiech ZA, Torres R, et al. Sequencing of 53,831 diverse genomes from the NHLBI TOPMed Program. Nature. 2021;590:290–9. 10.1038/s41586-021-03205-y. 33568819 10.1038/s41586-021-03205-y PMC7875770

[45] Bushnell B. BBMap. sourceforge.net/projects/bbmap/. Accessed 2 Sep 2024.

[46] Dobin A, Davis CA, Schlesinger F, Drenkow J, Zaleski C, Jha S, et al. STAR: ultrafast universal RNA-seq aligner. Bioinformatics. 2013;29:15–21. 10.1093/bioinformatics/bts635. 23104886 10.1093/bioinformatics/bts635 PMC3530905

[47] Andrews S. FastQC: A quality tool for high throughput sequence data. 2010. https://www.bioinformatics.babraham.ac.uk/projects/fastqc/. Accessed 2 Sep 2024.

[48] Wang L, Wang S, Li W. RSeQC: quality control of RNA-seq experiments. Bioinformatics. 2012;28:2184–5. 10.1093/bioinformatics/bts356. 22743226 10.1093/bioinformatics/bts356

[49] Ewels P, Magnusson M, Lundin S, Käller M. MultiQC: summarize analysis results for multiple tools and samples in a single report. Bioinformatics. 2016;32:3047–8. 10.1093/bioinformatics/btw354. 27312411 10.1093/bioinformatics/btw354 PMC5039924

[50] Pedregosa F, Varoquaux G, Gramfort A, Michel V, Thirion B, Grisel O, Blondel M, Prettenhofer P, Weiss R, Dubourg V, Vanderplas J, Passos A, Cournapeau D, Brucher M, Perrot M, Duchesnay D. Scikit-learn: machine learning in python. J Machine Learn Res. 2011;12:2825–30.

[51] Law CW, Alhamdoosh M, Su S, Dong X, Tian L, Smyth GK, Ritchie ME. RNA-seq analysis is easy as 1–2–3 with limma, Glimma and edgeR. F1000Res 2016. 10.12688/f1000research.9005.3. 10.12688/f1000research.9005.1 PMC4937821 27441086

[52] Robinson MD, McCarthy DJ, Smyth GK. edgeR: a Bioconductor package for differential expression analysis of digital gene expression data. Bioinformatics. 2010;26:139–40. 10.1093/bioinformatics/btp616. 19910308 10.1093/bioinformatics/btp616 PMC2796818

[53] Ritchie ME, Phipson B, Di Wu, Hu Y, Law CW, Shi W, Smyth GK. limma powers differential expression analyses for RNA-sequencing and microarray studies. Nucleic Acids Res. 2015;43:e47. 10.1093/nar/gkv007. 25605792 10.1093/nar/gkv007 PMC4402510

[54] Benjamini Y, Hochberg Y. Controlling the false discovery rate: a practical and powerful approach to multiple testing. J R Stat Soc Ser B Stat Methodol. 1995;57:289–300. 10.1111/j.2517-6161.1995.tb02031.x.

[55] Ongen H, Buil A, Brown AA, Dermitzakis ET, Delaneau O. Fast and efficient QTL mapper for thousands of molecular phenotypes. Bioinformatics. 2016;32:1479–85. 10.1093/bioinformatics/btv722. 26708335 10.1093/bioinformatics/btv722 PMC4866519

[56] Human genomics. The Genotype-Tissue Expression (GTEx) pilot analysis: multitissue gene regulation in humans. Science. 2015;348:648–60. 10.1126/science.1262110. 25954001 10.1126/science.1262110 PMC4547484

[57] Ashburner M, Ball CA, Blake JA, Botstein D, Butler H, Cherry JM, et al. Gene ontology: tool for the unification of biology. The gene ontology consortium. Nat Genet. 2000;25:25–9. 10.1038/75556. 10802651 10.1038/75556 PMC3037419

[58] Aleksander SA, Balhoff J, Carbon S, Cherry JM, Drabkin HJ, Ebert D, et al. The gene ontology knowledgebase in 2023. Genetics. 2023. 10.1093/genetics/iyad031. 36866529 10.1093/genetics/iyad031 PMC10158837

[59] Subramanian A, Tamayo P, Mootha VK, Mukherjee S, Ebert BL, Gillette MA, et al. Gene set enrichment analysis: a knowledge-based approach for interpreting genome-wide expression profiles. Proc Natl Acad Sci U S A. 2005;102:15545–50. 10.1073/pnas.0506580102. 16199517 10.1073/pnas.0506580102 PMC1239896

[60] Mootha VK, Lindgren CM, Eriksson K-F, Subramanian A, Sihag S, Lehar J, et al. PGC-1alpha-responsive genes involved in oxidative phosphorylation are coordinately downregulated in human diabetes. Nat Genet. 2003;34:267–73. 10.1038/ng1180. 12808457 10.1038/ng1180

[61] Wu T, Hu E, Xu S, Chen M, Guo P, Dai Z, et al. clusterProfiler 4.0: a universal enrichment tool for interpreting omics data. Innovation (Camb). 2021;2:100141. 10.1016/j.xinn.2021.100141. 34557778 10.1016/j.xinn.2021.100141 PMC8454663

[62] Liberzon A, Birger C, Thorvaldsdóttir H, Ghandi M, Mesirov JP, Tamayo P. The Molecular Signatures Database (MSigDB) hallmark gene set collection. Cell Syst. 2015;1:417–25. 10.1016/j.cels.2015.12.004. 26771021 10.1016/j.cels.2015.12.004 PMC4707969

[63] Ghoussaini M, Mountjoy E, Carmona M, Peat G, Schmidt EM, Hercules A, et al. Open Targets Genetics: systematic identification of trait-associated genes using large-scale genetics and functional genomics. Nucleic Acids Res. 2020;49:D1311–20. 10.1093/nar/gkaa840. 10.1093/nar/gkaa840 PMC7778936 33045747

[64] Mountjoy E, Schmidt EM, Carmona M, Schwartzentruber J, Peat G, Miranda A, et al. An open approach to systematically prioritize causal variants and genes at all published human GWAS trait-associated loci. Nat Genet. 2021;53:1527–33. 10.1038/s41588-021-00945-5. 34711957 10.1038/s41588-021-00945-5 PMC7611956

[65] Machiela MJ, Chanock SJ. LDlink: a web-based application for exploring population-specific haplotype structure and linking correlated alleles of possible functional variants. Bioinformatics. 2015;31:3555–7. 10.1093/bioinformatics/btv402. 26139635 10.1093/bioinformatics/btv402 PMC4626747

[66] Oelen R, de Vries DH, Brugge H, Gordon MG, Vochteloo M, Ye CJ, et al. Single-cell RNA-sequencing of peripheral blood mononuclear cells reveals widespread, context-specific gene expression regulation upon pathogenic exposure. Nat Commun. 2022;13:3267. 10.1038/s41467-022-30893-5. 35672358 10.1038/s41467-022-30893-5 PMC9174272

[67] Mullins N, Forstner AJ, O’Connell KS, Coombes B, Coleman JRI, Qiao Z, et al. Genome-wide association study of more than 40,000 bipolar disorder cases provides new insights into the underlying biology. Nat Genet. 2021;53:817–29. 10.1038/s41588-021-00857-4. 34002096 10.1038/s41588-021-00857-4 PMC8192451

[68] Multiple sclerosis genomic map implicates peripheral immune cells and microglia in susceptibility. Science 2019. 10.1126/science.aav7188. 10.1126/science.aav7188 PMC7241648 31604244

[69] Michailidou K, Lindström S, Dennis J, Beesley J, Hui S, Kar S, et al. Association analysis identifies 65 new breast cancer risk loci. Nature. 2017;551:92–4. 10.1038/nature24284. 29059683 10.1038/nature24284 PMC5798588

[70] Phelan CM, Kuchenbaecker KB, Tyrer JP, Kar SP, Lawrenson K, Winham SJ, et al. Identification of 12 new susceptibility loci for different histotypes of epithelial ovarian cancer. Nat Genet. 2017;49:680–91. 10.1038/ng.3826. 28346442 10.1038/ng.3826 PMC5612337

[71] Zhang H, Ahearn TU, Lecarpentier J, Barnes D, Beesley J, Qi G, et al. Genome-wide association study identifies 32 novel breast cancer susceptibility loci from overall and subtype-specific analyses. Nat Genet. 2020;52:572–81. 10.1038/s41588-020-0609-2. 32424353 10.1038/s41588-020-0609-2 PMC7808397

[72] Rashkin SR, Graff RE, Kachuri L, Thai KK, Alexeeff SE, Blatchins MA, et al. Pan-cancer study detects genetic risk variants and shared genetic basis in two large cohorts. Nat Commun. 2020;11:4423. 10.1038/s41467-020-18246-6. 32887889 10.1038/s41467-020-18246-6 PMC7473862

[73] Wang Z, McGlynn KA, Rajpert-De Meyts E, Bishop DT, Chung CC, Dalgaard MD, et al. Meta-analysis of five genome-wide association studies identifies multiple new loci associated with testicular germ cell tumor. Nat Genet. 2017;49:1141–7. 10.1038/ng.3879. 28604732 10.1038/ng.3879 PMC5490654

[74] Vijayakrishnan J, Qian M, Studd JB, Yang W, Kinnersley B, Law PJ, et al. Identification of four novel associations for B-cell acute lymphoblastic leukaemia risk. Nat Commun. 2019;10:5348. 10.1038/s41467-019-13069-6. 31767839 10.1038/s41467-019-13069-6 PMC6877561

[75] Went M, Sud A, Försti A, Halvarsson B-M, Weinhold N, Kimber S, et al. Identification of multiple risk loci and regulatory mechanisms influencing susceptibility to multiple myeloma. Nat Commun. 2018;9:3707. 10.1038/s41467-018-04989-w. 30213928 10.1038/s41467-018-04989-w PMC6137048

[76] Giambartolomei C, Vukcevic D, Schadt EE, Franke L, Hingorani AD, Wallace C, Plagnol V. Bayesian test for colocalisation between pairs of genetic association studies using summary statistics. PLoS Genet. 2014;10:e1004383. 10.1371/journal.pgen.1004383. 24830394 10.1371/journal.pgen.1004383 PMC4022491

[77] Wang B, Yin C, Yang X, Shi H, Zhang Z, Zhou J, Zhang P. Six genes associated with lymphatic metastasis in colon adenocarcinoma linked to prognostic value and tumor immune cell infiltration. Evid Based Complement Alternat Med. 2022;2022:4304361. 10.1155/2022/4304361. 36072412 10.1155/2022/4304361 PMC9444393

[78] Godel M, Morena D, Ananthanarayanan P, Buondonno I, Ferrero G, Hattinger CM, et al. Small nucleolar RNAs determine resistance to doxorubicin in human osteosarcoma. Int J Mol Sci. 2020. 10.3390/ijms21124500. 32599901 10.3390/ijms21124500 PMC7349977

[79] Luo L, Zhang J, Tang H, Zhai D, Huang D, Ling L, et al. LncRNA SNORD3A specifically sensitizes breast cancer cells to 5-FU by sponging miR-185-5p to enhance UMPS expression. Cell Death Dis. 2020;11:329. 10.1038/s41419-020-2557-2. 32382150 10.1038/s41419-020-2557-2 PMC7205983

[80] Menteş M, Yandım C. Identification of PPA1 inhibitor candidates for potential repurposing in cancer medicine. J Cell Biochem. 2023;124:1646–63. 10.1002/jcb.30475. 37733630 10.1002/jcb.30475

[81] Wang S, Wei J, Li S, Luo Y, Li Y, Wang X, et al. PPA1, an energy metabolism initiator, plays an important role in the progression of malignant tumors. Front Oncol. 2022;12:1012090. 10.3389/fonc.2022.1012090. 36505776 10.3389/fonc.2022.1012090 PMC9733535

[82] Takekawa M, Saito H. A family of stress-inducible GADD45-like proteins mediate activation of the stress-responsive MTK1/MEKK4 MAPKKK. Cell. 1998;95:521–30. 10.1016/S0092-8674(00)81619-0. 9827804 10.1016/s0092-8674(00)81619-0

[83] Jackson RA, Wu JS, Chen ES. C1D family proteins in coordinating RNA processing, chromosome condensation and DNA damage response. Cell Div. 2016;11:2. 10.1186/s13008-016-0014-5. 27030795 10.1186/s13008-016-0014-5 PMC4812661

[84] Siveen KS, Nizamuddin PB, Uddin S, Al-Thani M, Frenneaux MP, Janahi IA, et al. TRPV2: a cancer biomarker and potential therapeutic target. Dis Markers. 2020;2020:8892312. 10.1155/2020/8892312. 33376561 10.1155/2020/8892312 PMC7746447

[85] Hu S, Cai J, Fang H, Chen Z, Zhang J, Cai R. RPS14 promotes the development and progression of glioma via p53 signaling pathway. Exp Cell Res. 2023;423:113451. 10.1016/j.yexcr.2022.113451. 36535509 10.1016/j.yexcr.2022.113451

[86] Wang X, Yao S, Luo G, Zhou Y, Fang Q. Downregulation of RPS14 inhibits the proliferation and metastasis of estrogen receptor-positive breast cancer cells. Anticancer Drugs. 2021;32:1019–28. 10.1097/CAD.0000000000001112. 34261921 10.1097/CAD.0000000000001112

[87] Corcoran CA, He Q, Ponnusamy S, Ogretmen B, Huang Y, Sheikh MS. Neutral sphingomyelinase-3 is a DNA damage and nongenotoxic stress-regulated gene that is deregulated in human malignancies. Mol Cancer Res. 2008;6:795–807. 10.1158/1541-7786.MCR-07-2097. 18505924 10.1158/1541-7786.MCR-07-2097 PMC2642592

[88] Jonkers IH, Wijmenga C. Context-specific effects of genetic variants associated with autoimmune disease. Hum Mol Genet. 2017;26:R185–92. 10.1093/hmg/ddx254. 28977443 10.1093/hmg/ddx254 PMC5886469

[89] Fries GR, Vasconcelos-Moreno MP, Gubert C, Santos BTMQD, Da Rosa ALST, Eisele B, et al. Early apoptosis in peripheral blood mononuclear cells from patients with bipolar disorder. J Affect Disord. 2014;152–154:474–7. 10.1016/j.jad.2013.07.027. 24007785 10.1016/j.jad.2013.07.027

[90] Ponath G, Park C, Pitt D. The role of astrocytes in multiple sclerosis. Front Immunol. 2018;9:217. 10.3389/fimmu.2018.00217. 29515568 10.3389/fimmu.2018.00217 PMC5826071

[91] Dai N, Jones BDM, Husain MI. Astrocytes in the neuropathology of bipolar disorder: review of current evidence. Brain Sci. 2022. 10.3390/brainsci12111513. 36358439 10.3390/brainsci12111513 PMC9688542

[92] Potokar M, Jorgačevski J. Plectin in the central nervous system and a putative role in brain astrocytes. Cells. 2021. 10.3390/cells10092353. 34572001 10.3390/cells10092353 PMC8464768

[93] Chen S, Chen J, Hua X, Sun Y, Cui R, Sha J, Zhu X. The emerging role of XBP1 in cancer. Biomed Pharmacother. 2020;127:110069. 10.1016/j.biopha.2020.110069. 32294597 10.1016/j.biopha.2020.110069

[94] Chen X, Iliopoulos D, Zhang Q, Tang Q, Greenblatt MB, Hatziapostolou M, et al. XBP1 promotes triple-negative breast cancer by controlling the HIF1α pathway. Nature. 2014;508:103–7. 10.1038/nature13119. 24670641 10.1038/nature13119 PMC4105133

[95] Willis S, Villalobos VM, Gevaert O, Abramovitz M, Williams C, Sikic BI, Leyland-Jones B. Single gene prognostic biomarkers in ovarian cancer: a meta-analysis. PLoS One. 2016;11:e0149183. 10.1371/journal.pone.0149183. 26886260 10.1371/journal.pone.0149183 PMC4757072

[96] Panina Y, Germond A, Masui S, Watanabe TM. Validation of common housekeeping genes as reference for qPCR gene expression analysis during ips reprogramming process. Sci Rep. 2018;8:8716. 10.1038/s41598-018-26707-8. 29880849 10.1038/s41598-018-26707-8 PMC5992140

[97] Guo W, Zhang J, Zhou Y, Zhou C, Yang Y, Cong Z, et al. GPR160 is a potential biomarker associated with prostate cancer. Signal Transduct Target Ther. 2021;6:241. 10.1038/s41392-021-00583-7. 34168114 10.1038/s41392-021-00583-7 PMC8225807

[98] Jude JG, Spencer GJ, Huang X, Somerville TDD, Jones DR, Divecha N, Somervaille TCP. A targeted knockdown screen of genes coding for phosphoinositide modulators identifies PIP4K2A as required for acute myeloid leukemia cell proliferation and survival. Oncogene. 2015;34:1253–62. 10.1038/onc.2014.77. 24681948 10.1038/onc.2014.77 PMC4130659

[99] Li Y, Tu S, Zeng Y, Zhang C, Deng T, Luo W, et al. KLF2 inhibits TGF-β-mediated cancer cell motility in hepatocellular carcinoma. Acta Biochim Biophys Sin (Shanghai). 2020;52:485–94. 10.1093/abbs/gmaa024. 32318691 10.1093/abbs/gmaa024

[100] Li J, Jiang JL, Chen YM, Lu WQ. KLF2 inhibits colorectal cancer progression and metastasis by inducing ferroptosis via the PI3K/AKT signaling pathway. J Pathol Clin Res. 2023;9:423–35. 10.1002/cjp2.325. 37147883 10.1002/cjp2.325 PMC10397377

[101] Ohguchi H, Hideshima T, Bhasin MK, Gorgun GT, Santo L, Cea M, et al. The KDM3A-KLF2-IRF4 axis maintains myeloma cell survival. Nat Commun. 2016;7:10258. 10.1038/ncomms10258. 26728187 10.1038/ncomms10258 PMC4728406

