# Supplementary material for: Expression quantitative trait loci influence DNA damage-induced apoptosis in cancer
Source: BMC Genomics. 2024 Dec 2;25:1168. doi: 10.1186/s12864-024-11068-6 (PMC11613471; doi:10.1186/s12864-024-11068-6)
Supplement: Supplementary file 2 — Additional file 2: Supplementary figures. (pdf, Acrobat Reader https://www.adobe.com/de/acrobat.html). [file 12864_2024_11068_MOESM2_ESM.docx]

**Supplementary figures**

**Figure S1** Expression profiling of CD8^+^ T cells after carcinogen treatment

**Figure S2** Characteristics of identified eQTL

**Figure S3** Trait associated e^2^QTL

**a**


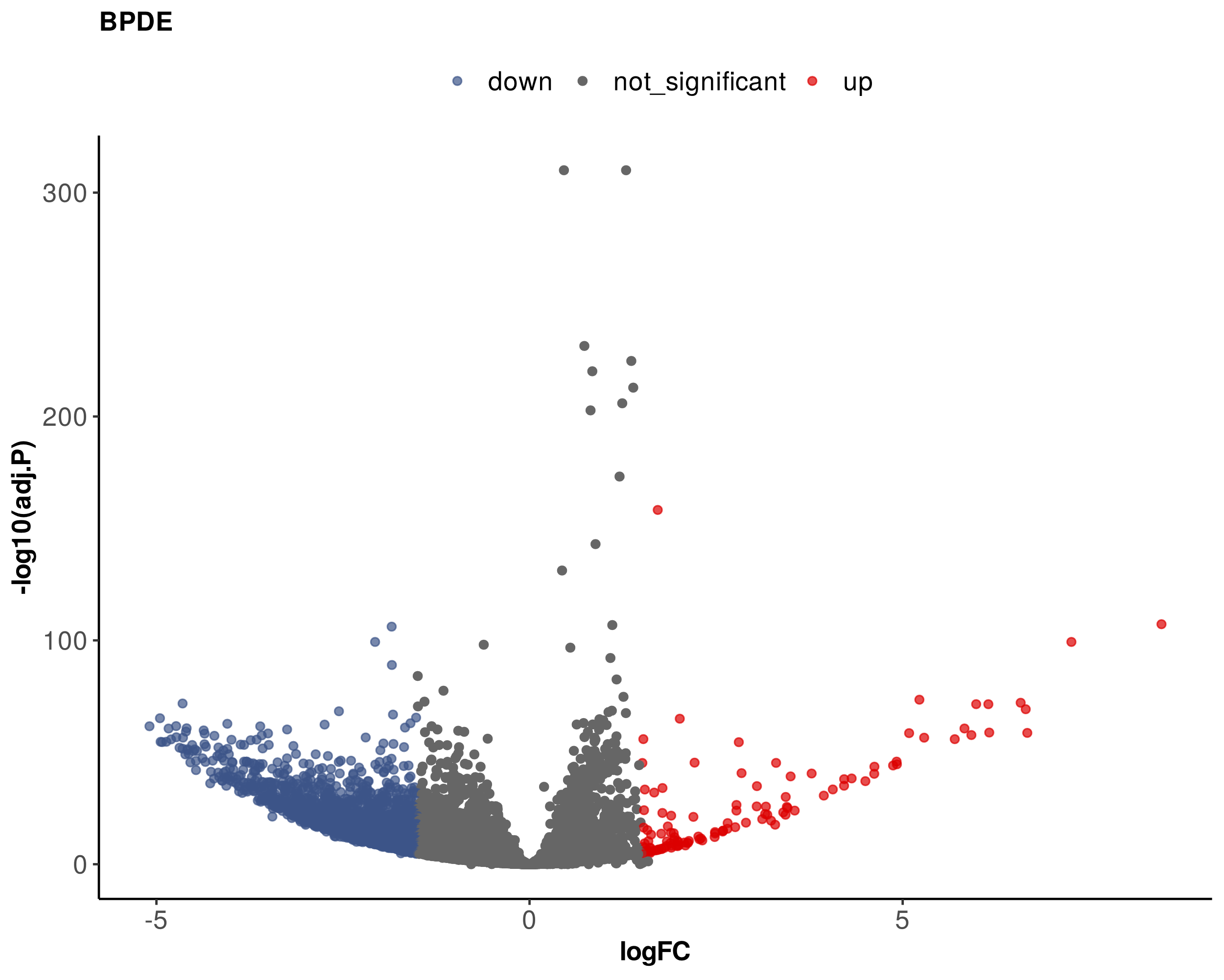

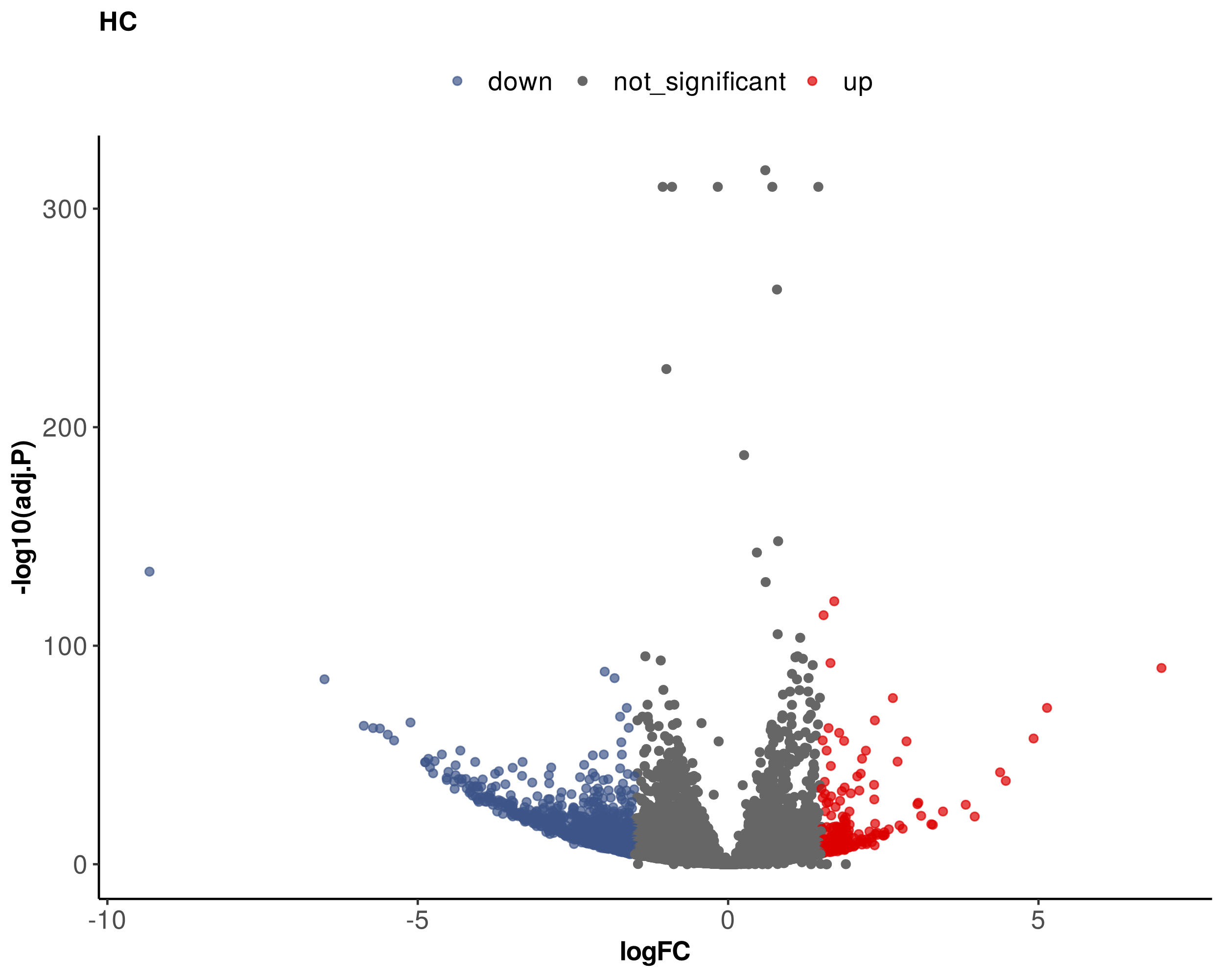

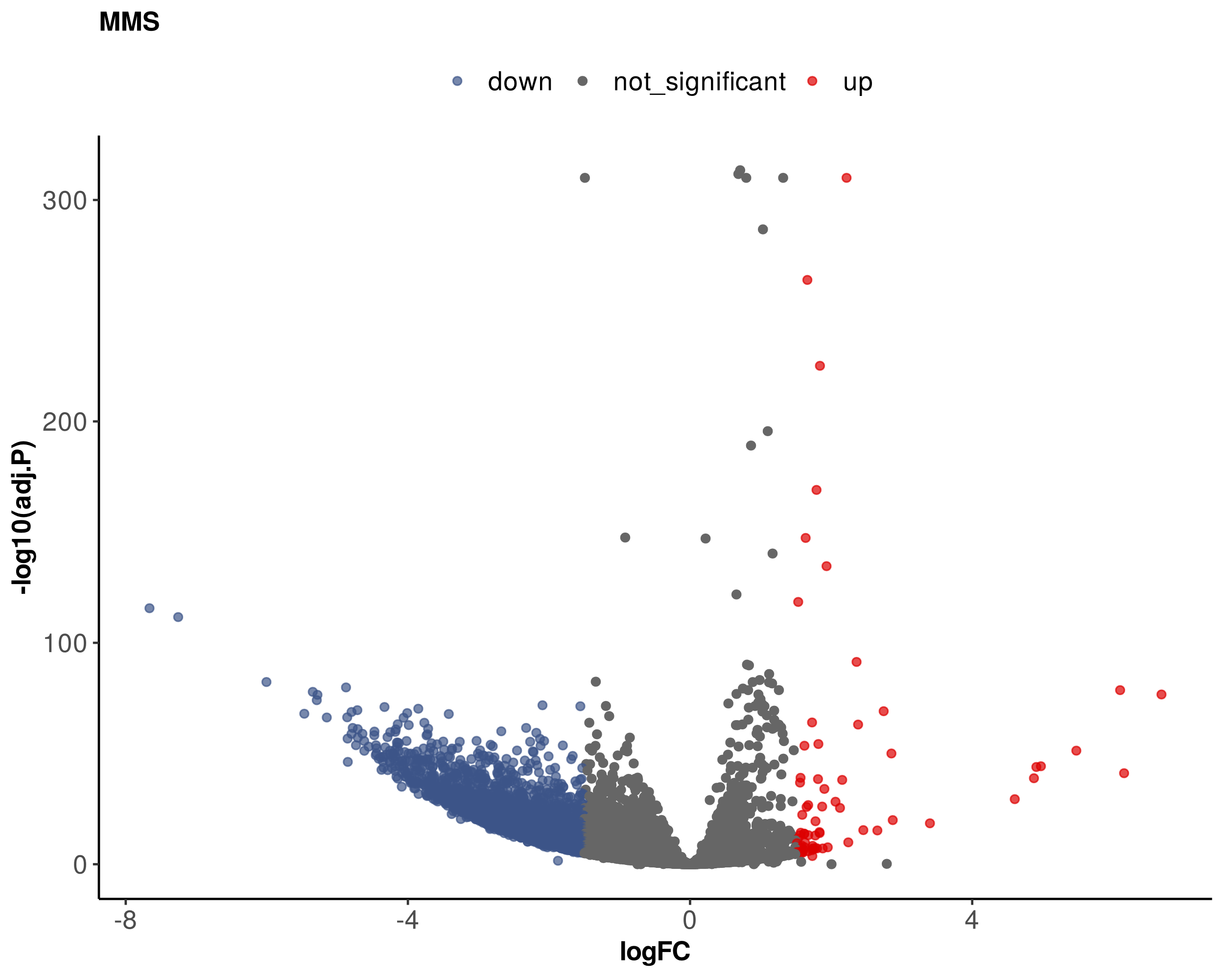

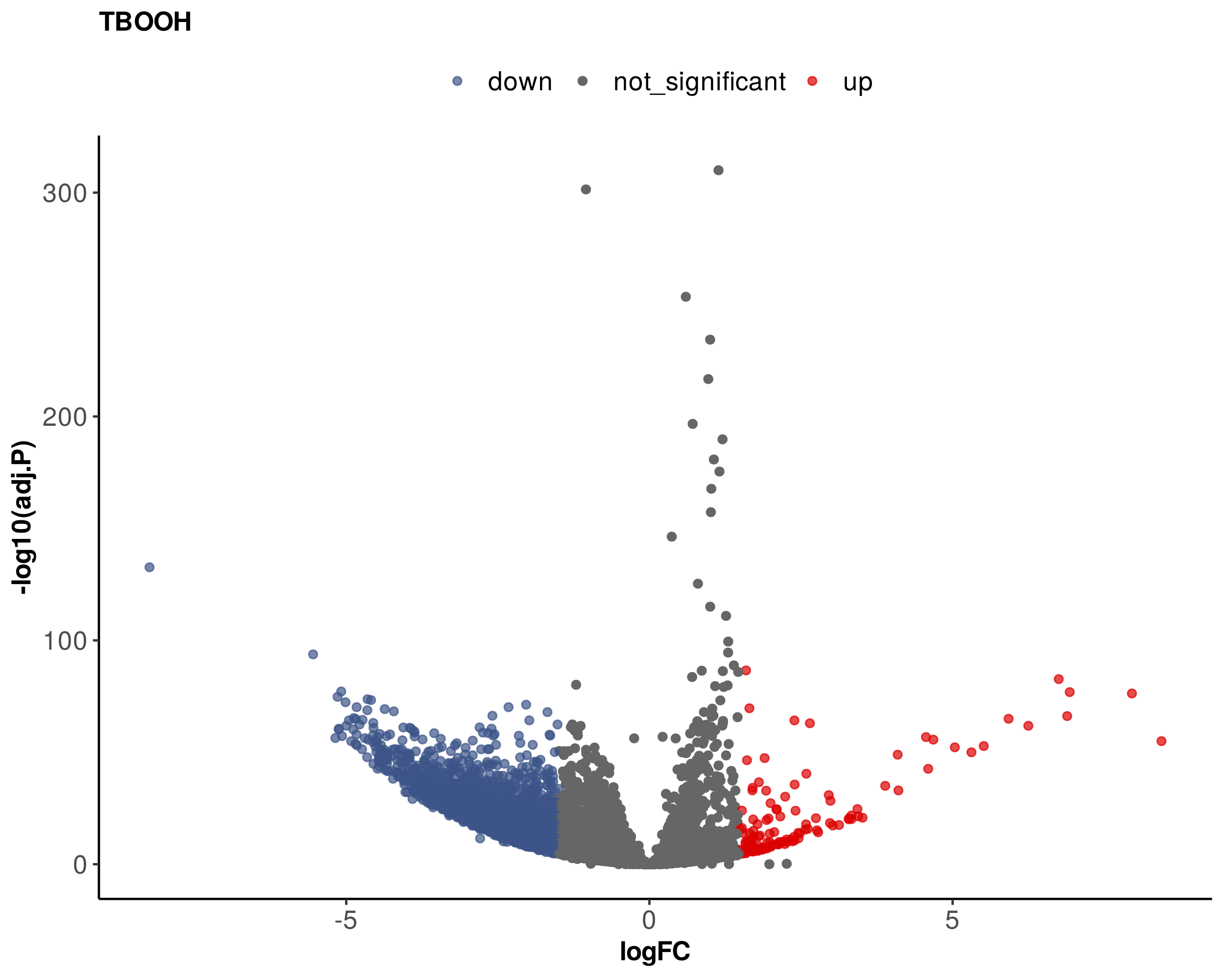

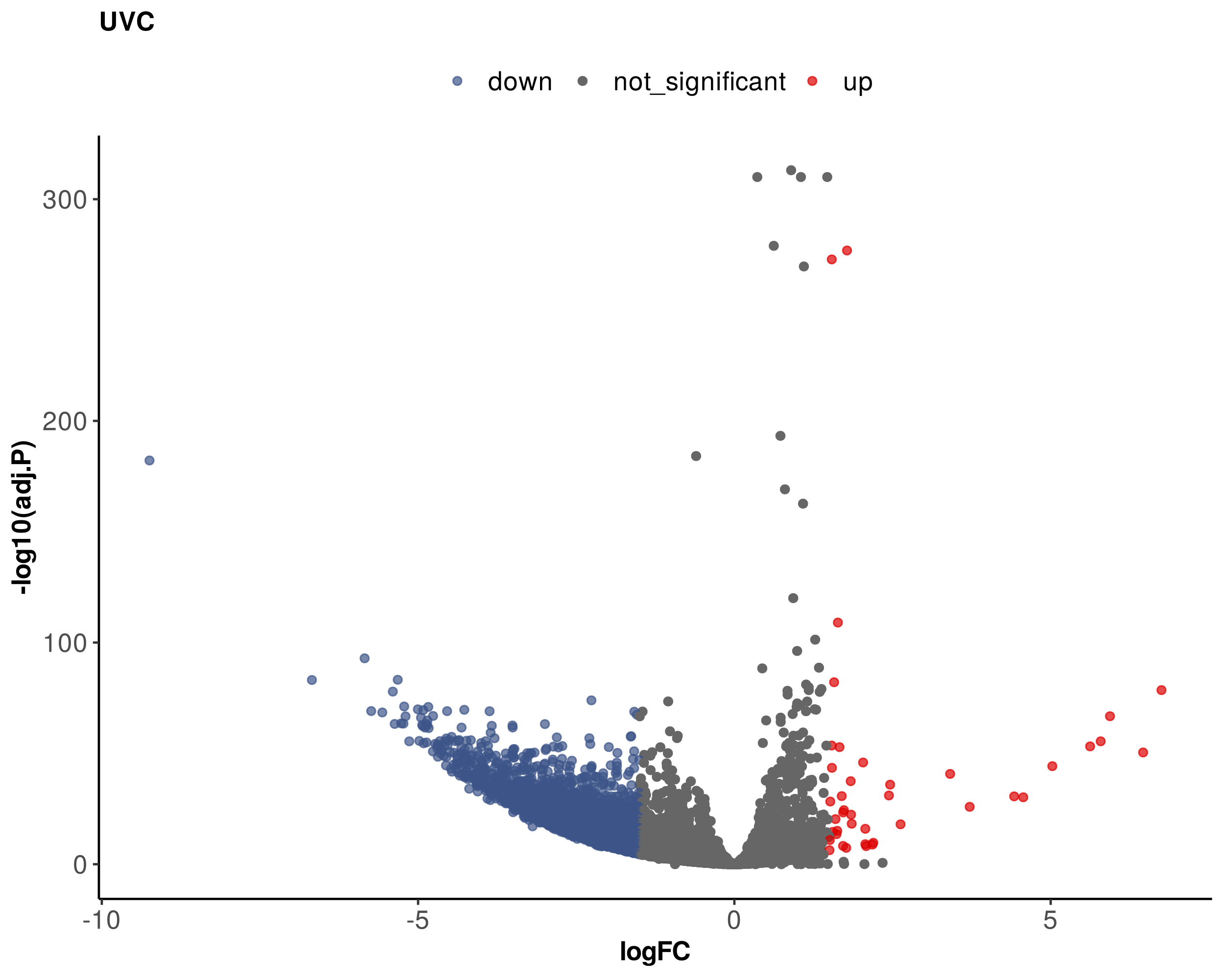


**b**


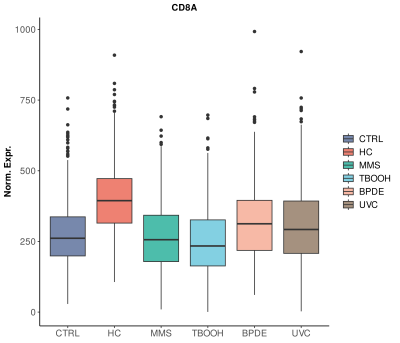

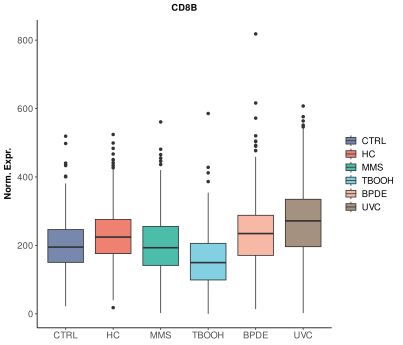

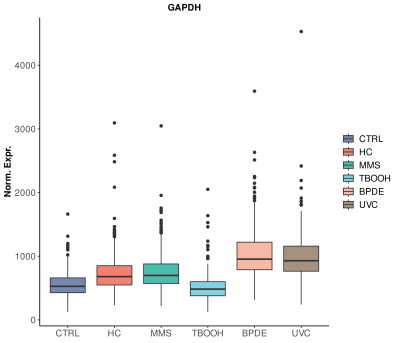

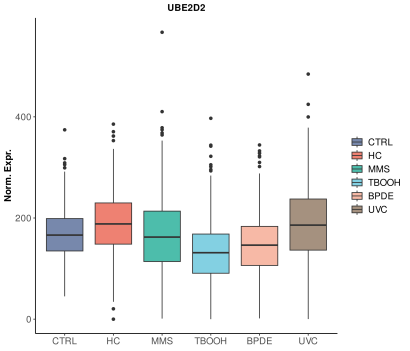


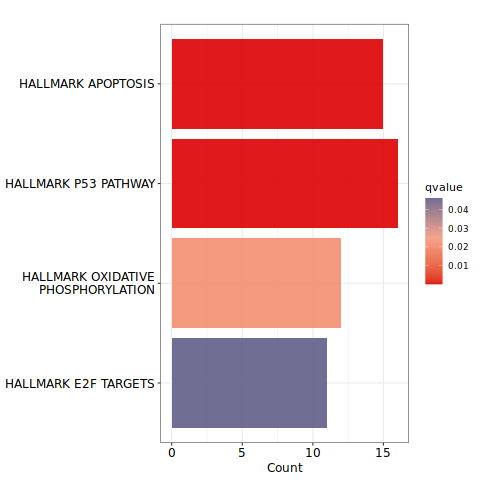

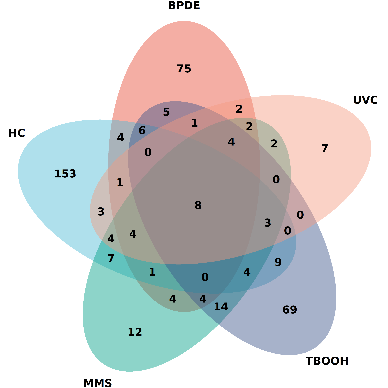


**d**

**c**

**SNORD3A**

**RNUV1-27**

**RNU4-2**

**SDR42E2**

**U2**

**ENSG00000279602**

**ENSG00000242299**

**ENSG00000213315**

**Figure S1** **Expression profiling of CD8^+^ T cells after carcinogen treatment** (a) Volcano plots of differential expression analysis. Significantly upregulated genes (adj. p < 0.05 and logFC > 1.5) are indicated in red and significantly downregulated genes (adj. p < 0.05 and logFC < -1.5) are indicated in blue. Multiple testing correction was performed using the Benjamini-Hochberg method. (b) Boxplots representing the normalized count distribution (TMM adjusted CPM) for *CD8A*, *CD8B*, *GAPDH* and *UBE2D*. (c) Venn diagram of significantly upregulated DEG. (d) GSEA of Molecular Signatures Database (MSigDB) hallmarks for all significantly upregulated DEG.

*BPDE - benzo(a)pyrene-7,8-diol-9,10-epoxide, CTRL – control, HC - 4-hydroxycyclophosphamide, MMS - Methyl-methanesulfonate, TBOOH - tert-butyl-hydroperoxide, UVC - ultraviolet radiation*


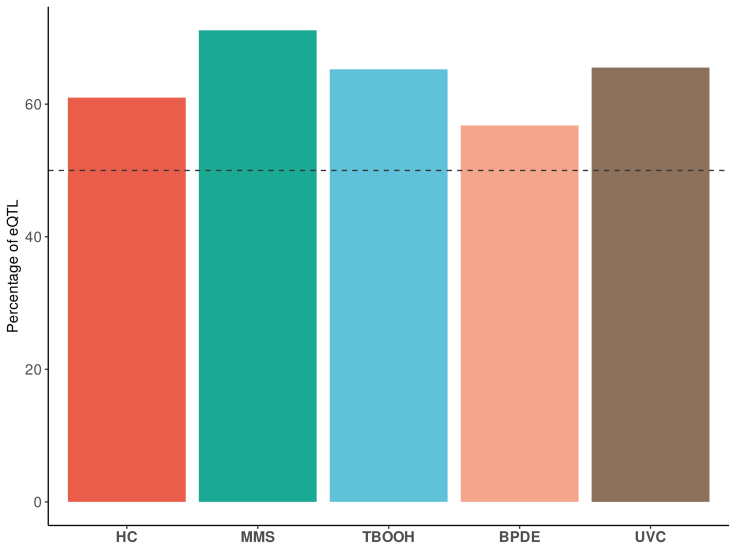

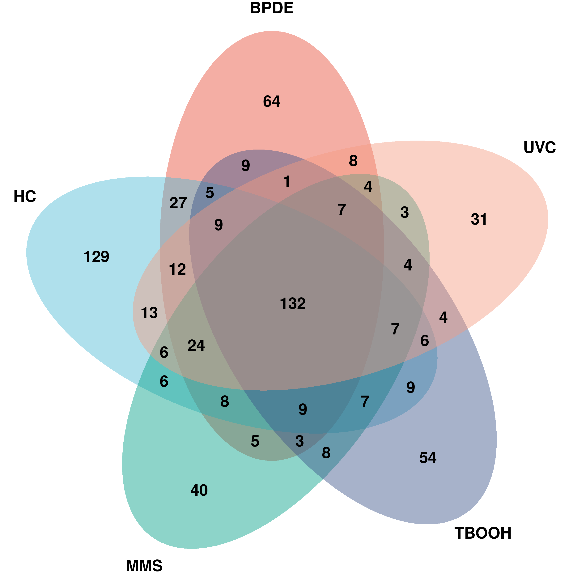


**a**

**b**

**Figure S2** **Characteristics of identified eQTL** (a) Venn diagram of significant eQTL eGenes. (b) Fraction of eQTL with reduced effect size after treatment for each stimulus.

*BPDE - benzo(a)pyrene-7,8-diol-9,10-epoxide, HC - 4-hydroxycyclophosphamide, MMS - Methyl-methanesulfonate, TBOOH - tert-butyl-hydroperoxide, UVC - ultraviolet radiation*


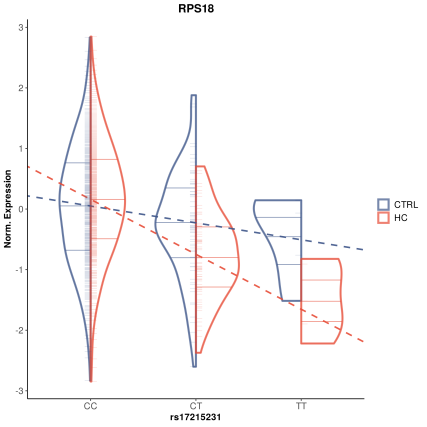

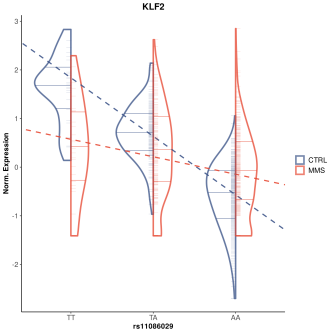

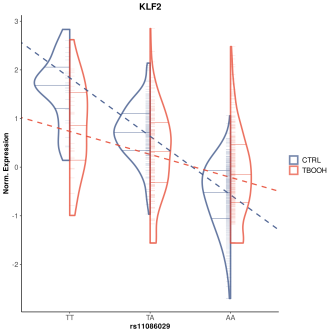

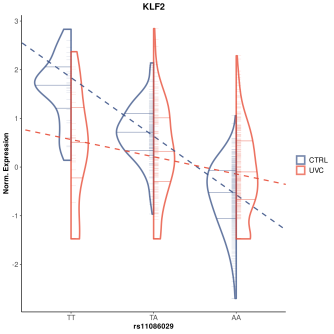

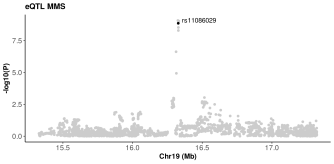

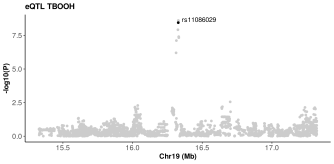

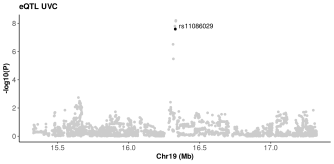


**a**

**b**

**Figure S3** **Trait associated e^2^QTL** (a) Violin plot of RPS18 and the GWAS lead variant for breast cancer before treatment (CTRL, blue) and after HC treatment (red). Individuals are indicated by small ticks and horizontal lines represent the median as well as the 25 % and 75 % quartiles in each violin. The dashed lines indicate the linear regression for the effect of the genotype on gene expression. (b) Violin plots for KLF2 and the GWAS lead variant for MM before treatment (CTRL, blue) and after treatment (red) with TBOOH (1), MMS (2) and HC (3). Scatter plots below show regional association of common variants in our eQTL data and the GWAS for MM. The lead variant of the MM GWAS is annotated and highlighted in black.

*CTRL – control, HC - 4-hydroxycyclophosphamide, MM – Multiple Myeloma, MMS - Methyl-methanesulfonate, TBOOH - tert-butyl-hydroperoxide, UVC - ultraviolet radiation*
